# Synthesis and Characterization of Sulfur Nanoparticles of *Citrus limon* Extract Embedded in Nanohydrogel Formulation: In Vitro and In Vivo Studies

**DOI:** 10.3390/gels9040284

**Published:** 2023-04-01

**Authors:** Hadia Baloch, Aisha Siddiqua, Asif Nawaz, Muhammad Shahid Latif, Syeda Qurbat Zahra, Suliman Yousef Alomar, Naushad Ahmad, Tarek M. Elsayed

**Affiliations:** 1 Gomal Centre of Biochemistry and Biotechnology, Gomal University, Dera Ismail Khan 29050, Pakistan; hadiabaloch2015@gmail.com (H.B.); draisha@gu.edu.pk (A.S.); syedaqurbatzahra@gmail.com (S.Q.Z.); 2 Advanced Drug Delivery Lab, Gomal Centre of Pharmaceutical Sciences, Faculty of Pharmacy, Gomal University, Dera Ismail Khan 29050, Pakistan; shahidlatif1710@gmail.com; 3 Doping Research Chair, Zoology Department, College of Science, King Saud University, Riyadh 11451, Saudi Arabia; syalomar@ksu.edu.sa; 4 Department of Chemistry, College of Science, King Saud University, Riyadh 11451, Saudi Arabia; anaushad@ksu.edu.sa; 5 Pharmaceutical Technology Department, Faculty of Pharmacy, Sultan Zainal Abidin University, Besut Kampus, Besut 22200, Malaysia; tarekelsayed@unisza.edu.my

**Keywords:** sulfur nanoparticles, *Citrus limon*, Gram-positive bacteria, Gram-negative bacteria, nanohydrogel

## Abstract

The study aimed to synthesize non-noxious, clean, reliable, and green sulfur nanoparticles (SNPs) from *Citrus limon* leaves. The synthesized SNPs were used to analyze particle size, zeta potential, UV–visible spectroscopy, SEM, and ATR-FTIR. The prepared SNPs exhibited a globule size of 55.32 ± 2.15 nm, PDI value of 0.365 ± 0.06, and zeta potential of −12.32 ± 0.23 mV. The presence of SNPs was confirmed by UV–visible spectroscopy in the range of 290 nm. The SEM image showed that the particles were spherical with a size of 40 nm. The ATR-FTIR study showed no interaction, and all the major peaks were preserved in the formulations. An antimicrobial and antifungal study of SNPs was carried out against Gram-positive bacteria (*Staph. aureus*, *Bacillus*), Gram-negative bacteria (*E. coli* and *Bordetella),* and fungal strains (*Candida albicans)*. The study showed that *Citrus limon* extract SNPs exhibited better antimicrobial and antifungal activities against *Staph. aureus*, *Bacillus*, *E. coli*, *Bordetella*, and *Candida albicans* at a minimal inhibitory concentration of 50 μg/mL. Different antibiotics were used alone and in combination with SNPs of *Citrus limon* extract to evaluate their activity against various strains of bacteria and fungal strains. The study showed that using SNPs of *Citrus limon* extract with antibiotics has a synergistic effect against *Staph.aureus*, *Bacillus*, *E. coli*, *Bordetella*, and *Candida albicans*. SNPs were embedded in nanohydrogel formulations for in vivo (wound healing) studies. In preclinical studies, SNPs of *Citrus limon* extract embedded within a nanohydrogel formulation (NHGF4) have shown promising results. To be widely used in clinical settings, further studies are needed to evaluate their safety and efficacy in human volunteers.

## 1. Introduction

Nanotechnology has the potential to revolutionize the pharmaceutical industry by enabling the development of new drug delivery systems that can improve the efficacy and safety of drugs [1]. Nanoparticles (NPs) have a wide range of potential applications in the pharmaceutical industry, including drug delivery, formulation, imaging, diagnosis, vaccines, and regenerative medicine [2]. NPs typically range in size from 1 to 100 nanometers (nm) [3].

In comparison to other processes, the green methods of nanoparticle preparation are those that use environmentally friendly and sustainable materials and processes, such as natural extracts, biopolymers, and water-based solvents, to synthesize nanoparticles [4]. These methods are gaining increasing attention in the field of nanotechnology due to their potential to reduce the environmental impact and toxicity associated with traditional nanoparticle synthesis methods, which often use hazardous chemicals and solvents [5].

Sulfur nanoparticles (SNPs) have potential applications in various fields, including agriculture and medicine, due to their unique properties, such as high surface area, catalytic activity, and biocompatibility [6]. The green synthesis of SNPs using plant-based materials is an environmentally friendly and sustainable approach that has gained increasing attention in recent years [7].

*Citrus limon*, commonly known as lemon, is a citrus fruit that contains various bioactive compounds, including sulfur compounds, which can be used for the synthesis of sulfur nanoparticles (SNPs) [8]. SNPs derived from *Citrus limon* have been reported to possess antibacterial activity against various types of bacteria, including Gram-positive bacteria and Gram-negative bacteria [9].

Gram-positive bacteria have a thick peptidoglycan layer in their cell walls, which makes them susceptible to disruption by certain antimicrobial agents [10]. SNPs have been shown to have the ability to penetrate the cell wall of Gram-positive bacteria and cause damage to the cell membrane, leading to cell death [11], while Gram-negative bacteria have an outer membrane that contains lipopolysaccharides, which can act as a barrier to prevent the entry of many antibacterial agents [12]. However, SNPs of *Citrus limon* can bind to the outer membrane of Gram-negative bacteria and disrupt the membrane integrity, leading to the leakage of cellular contents and, eventually, cell death [13]. They can also penetrate the cell wall and interact with intracellular components, such as DNA and proteins, leading to their inactivation [14].

A study showed that SNPs of *Citrus limon* have potential antimicrobial properties against fungal infection (*Candida albicans)* [15]. *Candida albicans* is a type of yeast that can cause infections in humans, especially in immunocompromised individuals [16]. SNPs of *Citrus limon* bind to the cell wall and membrane of *Candida albicans* and cause structural damage, leading to the leakage of cellular contents and, eventually, cell death [17]. They can also generate reactive oxygen species (ROS) that can cause oxidative damage to cellular components, including DNA, proteins, and lipids, leading to cell death. Moreover, SNPs can also inhibit the activity of enzymes that are essential for the growth and survival of *Candida albicans*, further contributing to their antifungal activity [18].

Nanohydrogels are a type of nanomaterial that is composed of cross-linked hydrophilic polymers, which can swell in water or other biological fluids [19]. They have unique properties that make them attractive for use in wound healing applications, such as their high water content, biocompatibility, and ability to encapsulate and deliver drugs [20]. The combination of SNPs of *Citrus limon* and nanohydrogels has been shown to have synergistic effects on wound healing [21]. In a study published in the International Journal of Nanomedicine, researchers developed a nanohydrogel loaded with silver nanoparticles of *Citrus limon* and evaluated its efficacy in treating infected wounds in rats. The results showed that the combination of these two materials was more effective in promoting wound healing and reducing bacterial growth than using either material alone [22].

The present study aimed to synthesize and characterize SNPs from *Citrus limon* for enhanced antibacterial activity against Gram-positive bacteria (*Staph. aureus* and *Bacillus*) and Gram-negative bacteria (*E. coli* and *Bordetella*), as well as antifungal (*Candida albicans*) activities. The SNPs of *Citrus limon* were embedded into a nanohydrogel for enhanced wound healing activities.

## 2. Results and Discussion

### 2.1. Characterization of Synthesized Sulfur Nanoparticles

Sulfur nanoparticles (SNPs) were prepared from the leaf extract of *Citrus limon* using the green synthesis method. The prepared SNPs were characterized and carried out for in vitro (antimicrobial and antifungal) activities and were then embedded in a nanohydrogel formulation for in vivo (wound healing) activities.

#### 2.1.1. Particle Size, Polydispersity Index, and Zeta Potential

**Figure 1:**
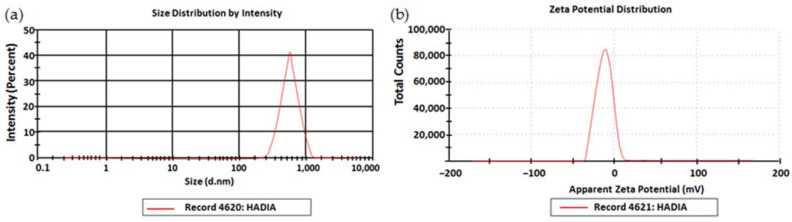
(**a**) Zeta size and (**b**) Zeta potential of SNPs.

The prepared SNPs exhibited a globule size of 55.32 ± 2.15 nm. The PDI value of the SNPs was 0.365 ± 0.06. The zeta potential value of the SNPs was −12.32 ± 0.23 mV. The zeta size and zeta potential of the SNPs are shown in Figure 1.

#### 2.1.2. UV−Visible Spectrophotometer

**Figure 2:**
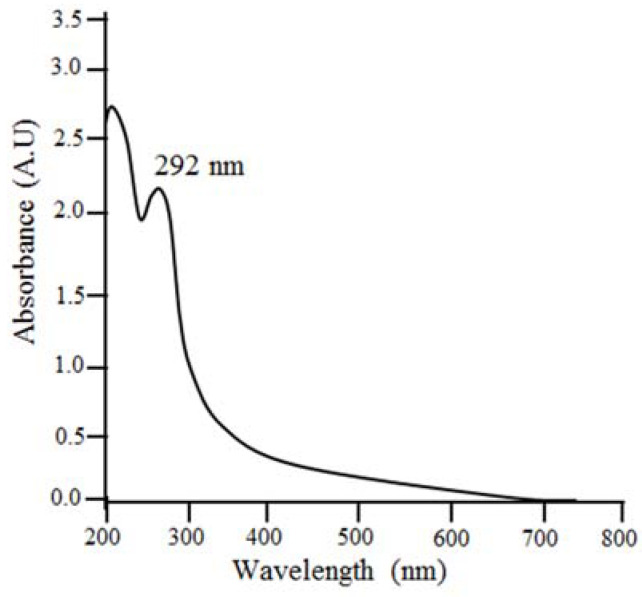
UV–visible spectroscopy of SNPs from leaves of *Citrus limon*.

UV–visible spectrophotometer analysis was used to verify SNPs’ synthesis of *Citrus limon*. After the reaction was completed, both nanoparticles were measured for their maximum absorption. *Citrus limon* nanoparticles showed a peak between 292 and 296 nm. Figure 2 shows an absorption peak at 292 nm. *A. indica*, *C. roseus*, *M. indica*, and *P. longifolia* were among four medicinal plants synthesized by Priti Paralikar and Mahendra Rai (2018) that exhibit absorption peaks at 292, 294, 296, and 294 nm, respectively [23].

#### 2.1.3. Scanning Electron Microscopy (SEM)

**Figure 3:**
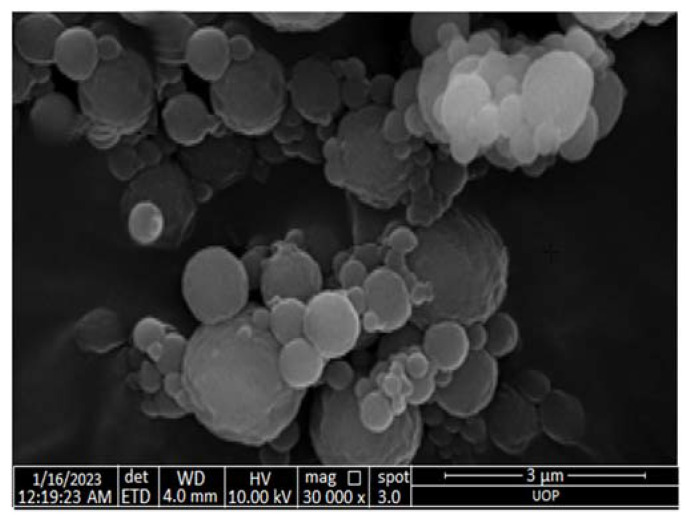
SEM image of SNPs synthesized from leaf extract of *Citrus limon*.

An SEM study was performed for sulfur nanoparticles synthesized from *Citrus limon* leaf extract (Figure 3). Based on the SEM micrograph, the particles were 40 nm in size. Except for a few ellipsoidal particles, the particles were almost spherical. The accumulation of small particles could be what caused the large particles. A study by Tripathi et al. (2018) indicated that SNPs synthesized from *F. benghalensis* leaf extract had a spherical shape and ranged in size from 25 to 120 nm [24].

#### 2.1.4. Attenuated Total Reflectance/Fourier Transform Infrared Spectroscopy (ATR-FTIR)

**Figure 4:**
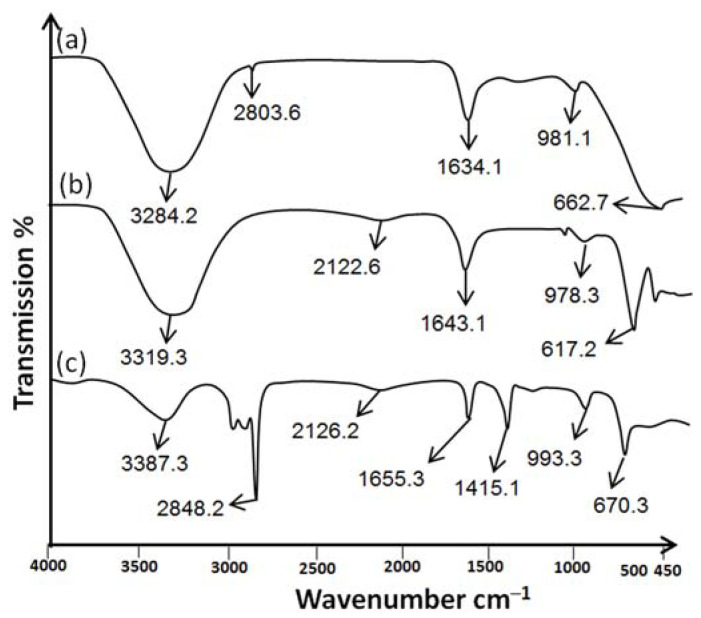
ATR-FTIR of (**a**) *Citrus limon* extract, (**b**) Sulfur nanoparticles, and (**c**) Nanohydrogel embedded with SNPs of *Citrus limon*.

Sulfur nanoparticles were investigated for the identification of functional groups using ATR-FTIR. The ATR-FTIR spectra of *Citrus limon* leaf extract showed a significant intensity peak at 3284.2 cm^−1^ with a wide transmittance band (3700–2800 cm^−1^). Figure 4a shows that there is a link between carboxylic acids and alcohols that are intermolecularly bound and their O–H starching vibrations. The weak bond is attributed to alkynes’ C≡C stretching vibrations at 2803.6 cm^−1^. The medium bond is attributed to the C=C stretching bond between alkenes at 1634.1 cm^−1^. The strong bonds between 981.1 cm^−1^ and 662.7 cm^−1^ are attributed to aromatic compounds bending vibration with benzene rings and C-H group stretching with halogen groups. In addition, Kumar et al. (2019) demonstrated that the functional groups of *Citrus limon* leaf extract showed the same major peak [25].

Figure 4b shows the ATR-FTIR spectrum of sulfur nanoparticles synthesized from *Citrus limon*. A high absorption peak was caused by the stretching vibration of phenols and alcohols at 3319.3 cm^−1^. A wide peak may result from symmetric and asymmetric stretching of the functional groups of aliphatic compounds. The amide carbonyl group of amide I and II may be substituted at 1643.1 cm^−1^. The peak at 978.3 cm^−1^ is associated with alcohol and amine stretching vibrations. As a result of aromatic molecules’ bending modes, there are extra peaks at 617.2 cm^−1^. Sulfur nanoparticles synthesized from pods of *Peltophorum pterocarpum* have the same broad peaks as those synthesized from *Citrus limon* leaf extract [26].

*Citrus limon* leaf extract embedded in nanohydrogel formulation (NHGF4) is shown in Figure 4c. The characteristic peaks of SNPs of NHGF4 were slightly shifted and/or reduced in intensity at 3387.3 cm^−1^. The weak bond at 2848.2 cm^−1^ is caused by the alkynes’ C≡C stretching vibrations. Alkenes C=C stretching was observed at 1655.3 cm^−1^. The stretching of aliphatic functional groups, both symmetric and asymmetric, may result in a broad peak at 2126.2 cm^−1^. The stretching of the carboxyl group was shown at 1415.1 cm^−1^. The C–O and C–N stretching vibrations of alcohol and amine were shown at peaks of 993.3 cm^−1^, respectively. The bending modes of aromatic molecules cause extra peaks at 670.3 cm^−1^. The electrostatic interaction or hydrogen bonding of nanohydrogel was due to the slight change in the peaks at 1655.3 cm^−1^ and 670.3 cm^−1^. According to Jaiswal et al. (2019), hydrogels loaded with nanoparticles showed the same peaks with minor differences [27]. The nanohydrogel formulation (NHGF4) showed no interaction in the ATR-FTIR study, and all major peaks were preserved, proving its suitability for further applications. 

### 2.2. In Vitro Studies

#### Antibacterial and Antifungal Activity

Gram-positive bacteria (*Staph. aureus* and *Bacillus*), Gram-negative bacteria (*E. coli* and *Bordetella*), and a fungal strain (*Candida albicans*) were treated with SNPs, various antibiotics, and a combination of SNPs with antibiotics using the well diffusion method.

The Gram-positive bacteria (*Staph. aureus* and *Bacillus*) were treated with SNPs, erythromycin thiocyanate (ET), and a combination of SNPs with erythromycin thiocyanate (SNPsET). The SNPs showed antimicrobial activity against *Staph.aureus* and *Bacillus* due to the presence of *Citrus limon* extract. The SNPs of *Citrus limon* extract are attributed to its ability to disrupt the cell membranes of bacteria, leading to bacterial cell death. Erythromycin thiocyanate also showed antimicrobial activity. Erythromycin thiocyanate works by inhibiting the growth and replication of bacteria by binding to the 50S ribosomal subunit, thereby preventing the formation of peptide bonds during protein synthesis. The combination of SNPs and erythromycin thiocyanate have shown synergistic effects against *Staph. aureus* and *Bacillus.* SNPs disrupt the bacterial cell membrane, while erythromycin thiocyanate inhibits bacterial protein synthesis. The combination of SNPs and erythromycin thiocyanate worked together to synergistically enhance the overall antimicrobial effect.

The Gram-negative bacteria (*E. coli* and *Bordetella*) were treated with SNPs, neomycin sulfate (NS), and a combination of SNPs with neomycin sulfate (SNPsNS). The SNPs showed antimicrobial activity against *E. coli* and *Bordetella* due to *Citrus limon* extract. The SNPs of *Citrus limon* extract are attributed to its ability to disrupt the cell membrane of bacteria, leading to bacterial cell death. Neomycin sulfate showed antimicrobial activity. Neomycin sulfate specifically targets the outer membranes of Gram-negative bacteria, which is rich in negatively charged lipopolysaccharides. These lipopolysaccharides are thought to help facilitate the uptake of the neomycin molecule into the cell, allowing it to bind to the bacterial ribosome and inhibit protein synthesis. The SNPs and neomycin sulfate combinations have synergistic effects against *E. coli* and *Bordetella*. The SNPs potentially disrupt the bacterial cell membrane and interfere with key metabolic processes, while neomycin sulfate inhibits protein synthesis, leading to cell death.

The fungal strain (*Candida albicans*) was treated with SNPs, Amphotericin B (AB), and a combination of SNPs with Amphotericin B (SNPsAB). The SNPs showed antifungal activity against *Candida albicans* due to the presence of *Citrus limon* extract. The antifungal activity of *Citrus limon* extract might be due to the presence of a high content of essential oils, including limonene and citral, which have been shown to have antifungal activities. Additionally, SNPs have synergistic antifungal effects on *Candida albicans*. Amphotericin B (AB) showed antifungal activity. Amphotericin B (AB) binds to the fungal cell membrane and disrupts its integrity, leading to the leakage of essential cellular components and eventual cell death. Combining SNPs with Amphotericin B (SNPsAB) has shown a synergistic effect against *Candida albicans.* This activity is thought to be due to the ability of SNPs to penetrate and disrupt the fungal cell wall, leading to cell death, while Amphotericin B (AB) is an antifungal medication that works by binding to the fungal cell membrane and disrupting its integrity, leading to the leakage of essential cellular components and eventual cell death.

**Figure 5:**
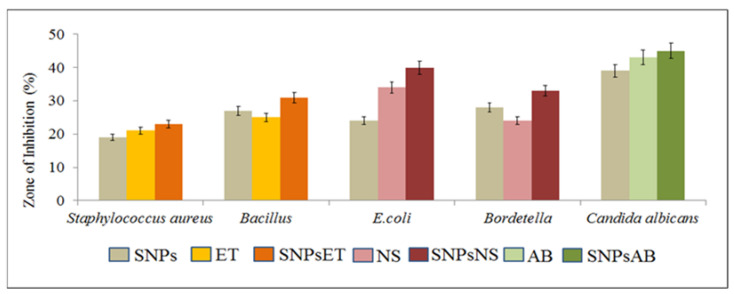
Antimicrobial and antifungal activity of SNPs of Citrus limon extract against different strains. Note: SNPs (sulfur nanoparticles), ET (erythromycin thiocyanate), SNPsET (sulfur nanoparticles embedded with erythromycin thiocyanate), NS (neomycin sulfate), SNPsNS (sulfur nanoparticles embedded with neomycin sulfate), AB (Amphotericin B), SNPsAB (sulfur nanoparticles embedded with Amphotericin B).

**Figure 6:**
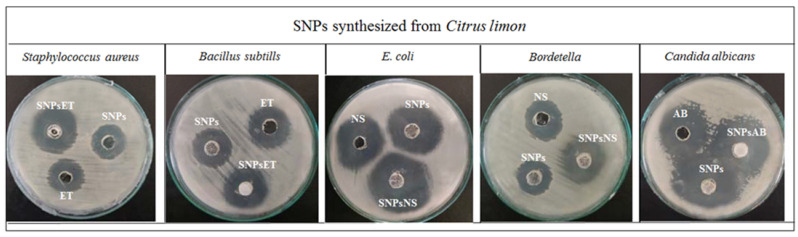
Antimicrobial and antifungal activity of SNPs of *Citrus limon* extract against different strains.

The overall findings of the study showed that the combination of antibiotics with SNPs synergistically enhanced their antimicrobial and antifungal activities against Gram-positive (*Staph. aureus* and *Bacillus)* and Gram-negative (*E. coli* and *Bordetella*) and fungal strains (*Candida albicans*), as shown in Figure 5 and Figure 6.

### 2.3. Characterization of Nanohydrogel

#### 2.3.1. Organoleptic Evaluation of Nanohydrogel

The organoleptic evaluation of nanohydrogels is essential to ensure they are suitable for their intended use and meet quality standards. Hydrogels’ effectiveness is affected by their pH. The pH range of SPNs-embedded nanohydrogel formulations (NHGF4) must be suitable for the skin [28]. Optimized formulations were adjusted to normal pH by adding NaOH dropwise under continuous stirring. Table 1 shows that NHGF4 had an average pH of 5.0–6.0, in accordance with human skin pH. The odor of the NHGF4 was evaluated and was found to be good enough to be used further. The color of the NHGF4 was also evaluated physically and was found to be off-white. NHGF4 showed no phase separation. A homogeneity test was conducted to ensure that the components in the formulations were uniform. The homogeneity of NHGF4 was good. Under a microscope, no lumps were found, and no particles were visible in the formulation smear. According to Krambeck (2021), the uniformity and consistency of nanohydrogels can be evaluated based on their organoleptic properties [29].

#### 2.3.2. Viscosity of Nanohydrogels

**Figure 7:**
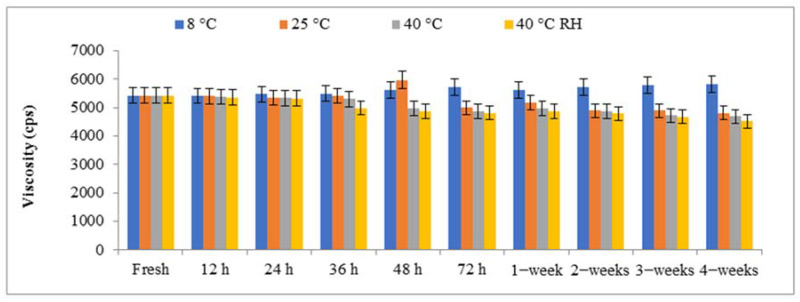
Viscosity of SNP nanoparticles of *Citrus limon* extract embedded in nanohydrogel formulation (NHGF4) at various temperatures (8 °C, 25 °C, 40 °C, and 40 °C RH), respectively.

Hydrogel formulations are highly dependent on viscosity. It is always inversely proportional to the amount of diffusion if it is a rate-limiting step. Increasing viscosity often results in a decrease in the medication release rate. With the addition of the liquid phase, the viscosity of the hydrogel is reduced [30]. For this study, different shear stresses were applied at different revolutions per minute, i.e., 6 revolutions per minute, 12 revolutions per minute, 30 revolutions per minute, and 60 revolutions per minute, to determine the optimum viscosity. The hydrogel’s viscosity was greatly reduced when the shear stress was increased. The hydrogel composition was measured using the NDJ-5 s viscometer using spindle number two. In the case of hydrogels, viscosity is an important factor for their application. High viscosities result in spreadability problems, whereas low viscosities result in surface flow [31]. Figure 7 shows that the viscosity of NHGF4 was within acceptable limits on the skin. Increasing the surfactant concentration increases the system’s yield value and viscosity. A network of oil droplets, micelles, and surfactant molecules forms when the concentration of the external phase rises. Furthermore, the gap between the yield value and disperse phase is close when the network is denser and the viscosity is higher. As temperature increases, viscosity decreases, and when temperature decreases, velocity increases [32].

### 2.4. Skin Irritation Test

**Figure 8:**
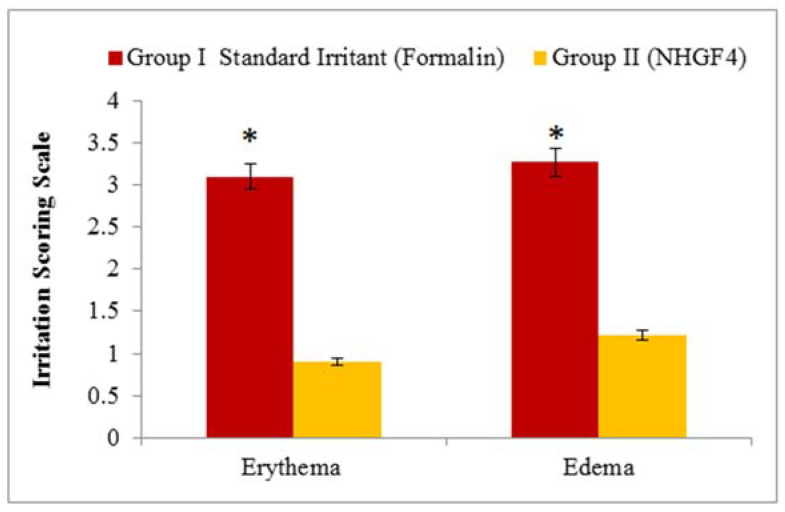
Skin irritation studies: erythema and edema. Data expressed as mean ± SD; *n* = 3. One-way ANOVA followed by post hoc Tukey test; * *p* < 0.05).

A potential skin irritation study was conducted on NHGF4, as shown in Figure 8. Skin changes such as inflammation, swelling, or other symptoms were not observed after using the formulations. In conclusion, the nanohydrogel formulation (NHGF4) was not irritating and can be applied topically.

### 2.5. In Vivo Studies

**Figure 9:**
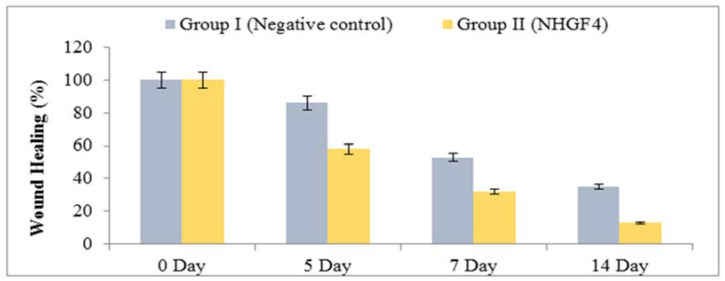
In vivo wound healing activity of SNPs embedded with NHGF4.

**Figure 10:**
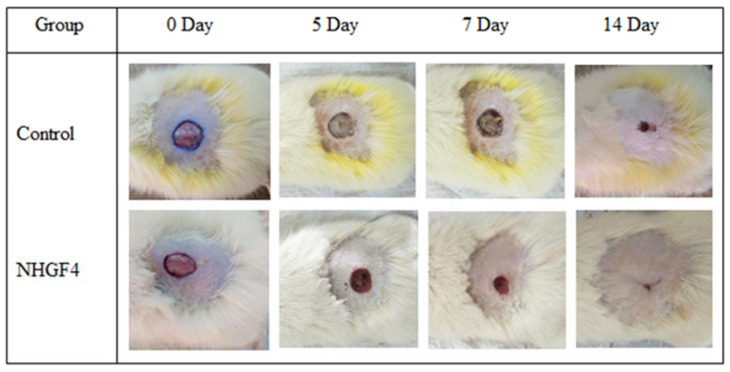
Image showing in vivo (wound healing) activity of Negative Control (Group I) and SNPs embedded with NHGF4 (Group II).

In vivo studies were performed to investigate the effectiveness of NHGF4 in wound healing. Albino male rats were used to study the effect of NHGF4 on incision wound healing. Group I consisted of rats that were negative controls, and Group II consisted of rats that were treated with NHGF4. Figure 9 and Figure 10 showed the % reductions in wound areas for Groups I and II, respectively.

In a study comparing a wound healing activity of a treatment of Group I (negative control) to Group II containing SNPs of *Citrus limon* extract embedded in a nanohydrogel formulation (NHGF4), it was observed that Group II showed significant improvement in wound healing over a 14-day period compared to Group II. During the first 24–48 h, the wounds in both groups exhibited similar characteristics, including redness, swelling, and pain. However, from the third day onwards, Group II (NHGF4) showed a marked reduction in these symptoms, indicating faster healing. By the end of the first week, the treatment group had developed a scab or a thin layer of tissue over the wound, indicating the initiation of the healing process.

Over the second week, the wounds continued healing in Group II (NHGF4), with the scab becoming more substantial. In contrast, the wounds in Group I (negative control) continued to exhibit inflammation, and the healing process was slower, with no sign of scab formation. By the end of the two weeks, Group II (NHGF4) showed complete wound healing, with the scabs falling off and the skin looking normal, while Group I (negative control) still had open wounds with visible signs of inflammation. Overall, the study showed that the treatment had a positive impact on wound healing, accelerating the process and reducing the time required for completing healing.

Sulfur in topical treatments induces hydrogen sulfide and keratosis in the skin through its interaction with keratinocytes. Several histological changes can also be induced by sulfur, including hyperkeratosis, acanthosis, and the dilation of dermal arteries [33]. SNPs of *Citrus limon* extract embedded in nanohydrogel formulation (NHGF4) has potent antioxidant properties that help neutralize free radicals and reduce oxidative stress in the wound site. This can help promote healing by reducing inflammation and preventing further damage to the tissue. The *Citrus limon* extract contains anti-inflammatory compounds, such as flavonoids and limonene [34]. These compounds can help reduce inflammation in the wound site and promote healing. The high acidity of *Citrus limon* extract creates an unfavorable environment for bacteria to grow [35]. Moreover, *Citrus limon* extract can stimulate collagen production, which is essential for wound healing, as collagen is a protein that helps rebuild damaged tissue and create new skin [36]. SNPs embedded in a nanohydrogel formulation provide a sustained release of therapeutic agents, which can improve the efficacy of the treatment and reduce the need for frequent applications.

Overall, using SNPs of *Citrus limon* extract embedded in a nanohydrogel formulation (NHGF4) has shown promising results in preclinical studies. However, further research is needed to assess their safety and efficacy in humans before they can be widely used in clinical settings.

### 2.6. Stability Test

ICH guidelines were followed for the stability analysis. An assessment of NHGF4’s physical appearance was conducted using a stability test. After 60 days, NHGF4 parameters such as odor, color, phase separation, homogeneity, and all other significant parameters were monitored. This process must be undertaken to determine whether formulations are either changing or remaining unchanged in appearance or effectiveness [37]. At low and exaggerated temperatures of 4 ± 2 °C and 40 ± 2 °C, respectively, NHGF4 passed the stability test in this study. Physically, NHGF4 remained the same color and smell. In addition, the NHGF4 formulation did not exhibit any phase separation over a predetermined time frame. In a study, it was noted that pH and temperature have an influence on the rate of nanohydrogel degradation. A nanohydrogel loses its structure and propensity when temperature and pH are increased [38]. The formulation (NHGF4) was found to maintain its structural integrity after 60 days (Table 2).

## 3. Conclusions

Using sulfur nanoparticles of *Citrus limon* showed promising results in inhibiting the growth of *Staphylococcus aureus*, *Bacillus*, *E. coli*, *Bordetella*, and *Candida albicans*. Using *Citrus limon* as a source of sulfur nanoparticles is advantageous, as it is easily accessible and possesses various bioactive compounds with potential therapeutic effects. Furthermore, using sulfur nanoparticles of *Citrus limon* embedded in a nanohydrogel formulation (NHGF4) showed potential in promoting wound healing activity in rats. NHGF4 exhibited significant wound closure indicating its effectiveness in accelerating the healing process. The nanohydrogel matrix also provided a suitable cell proliferation and tissue regeneration environment. Therefore, the study concluded that the sulfur nanoparticles of *Citrus limon* embedded in a nanohydrogel formulation could be a promising candidate for wound healing applications. However, further studies are required to investigate the safety and efficacy of the formulation in humans and optimize the dosage and application methods for clinical use.

## 4. Materials and Methods

### 4.1. Materials

Sodium thiosulfate pentahydrate (Na_2_S_2_O_3_·5H_2_O) and HCl were purchased from Merck Darmstadt, Germany. Distilled water was obtained through the Milli-Q water purification system of the Gomal Centre of Biochemistry and Biotechnology, Gomal University, Dera Ismail Khan, KP, Pakistan. Carbopol and triethanolamine were purchased from Sigma-Aldrich, St. Louis, CA, USA. Nutrient agar and nutrient broth were procured from Oxide, UK. Leaves of *Citrus limon* were collected from the Botanical Garden, Faculty of Agriculture, Gomal University, Dera Ismail Khan, Pakistan. Erythromycin thiocyanate was purchased from Sigma-Aldrich, St. Louis, CA, USA. Amphotericin B and neomycin sulfate were purchased from Dow chemical company, Wiesbaden, Germany. Strains of *Escherichia coli* (ATCC 25922), *Staphylococcus aureus* (ATCC 6538), *Bacillus subtilis* (ATCC 6633), *Bordetella* (ATCC4617), and *Candida albicans* (ATCC 10231) were taken from Bio-labs, Islamabad, Pakistan.

### 4.2. Plant Extract Formation

After collecting *Citrus limon* leaves, they were rinsed under running tap water to remove dirt. To remove any surface moisture from the leaves, they were dried at room temperature after washing. In a mechanical blender, the leaves were chopped after drying. A mixture of 10 g of chopped leaves in 100 mL of distilled water was prepared. At 90 °C, the mixture was boiled for 20 min in a water bath. A Whatman filter paper was used to filter this combination after it had been cooled down to ambient temperature. Centrifugation of the obtained mixture for 10 min at 7000 rpm was done to remove biomaterials with high density. The supernatant was refrigerated and used as a plant extract for later use [39].

### 4.3. Synthesis of Sulfur Nanoparticles

**Figure 11:**
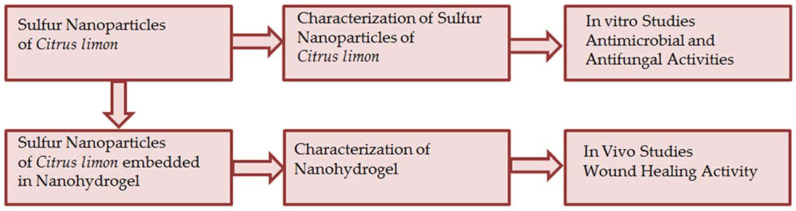
Scheme of the experimental procedure.

The method used by K Khairan et al. (2023) for synthesizing sulfur nanoparticles was slightly modified [40]. A total of 10 mL of plant extract was taken, and 5 g of sodium thiosulfate pentahydrate (Na_2_S_2_O_3_·5H_2_O) was added and stirred with a magnetic stirrer for 20 min. The extract was then agitated while 10 mL of 10% HCl was added dropwise using a dropper. To ensure homogeneous sulfur precipitation, continuous stirring was adopted. For 10 min, the mixture was centrifuged at 5000 rpm. It is essential that the sulfur precipitates are thoroughly cleaned after centrifugation by being washed three times in double-distilled water, with the supernatant discarded following the final washing. Refrigeration was used to dry sulfur nanoparticles after they had been collected. The sulfur nanoparticles were stored at room temperature after drying for further studies. The sulfur nanoparticles of *Citrus limon* were further characterized for in vitro, antimicrobial, and antifungal activities. Furthermore, these prepared SNPs were embedded into Nanohydrogel formulation for in vivo, and wound healing activities (Figure 11). 

### 4.4. Characterization of Synthesized Sulfur Nanoparticles

#### 4.4.1. Particle Size and Zeta Potential

NPs were characterized using photon correlation spectroscopy (Malvern Zetasizer Nano series Nano-S and Nano-Z, Malvern Instruments Ltd., Worcestershire, UK). SNPs were dispersed in deionized water for 5 mL and analyzed on Zetasizer. Zeta potential was measured using special tubes. All experiments were run in triplicate; mean ± SD, *n* = 3 [41].

#### 4.4.2. UV–Visible Spectroscopy

To characterize already synthesized sulfur nanoparticles with the scanning range of 200–800 nm, UV–visible spectroscopy (Shimadzu 1601, Kyoto, Japan) was employed. Sulfur nanoparticles were confirmed by this experiment [42].

#### 4.4.3. ATR-FTIR Analysis

ATR-FTIR (L1600300, PerkinElmer, 940 Winter Street Waltham, MA, USA), was used to analyze biomolecules responsible for ions reduction and capping. Sulfur nanoparticles, sodium thiosulfate pentahydrate, and *Citrus limon* leaf extract was studied using ATR-FTIR. The ATR-FTIR spectra were obtained between 4000–450 cm^−1^ [43].

#### 4.4.4. Scanning Electron Microscopy (SEM)

The size and shape of the synthesized nanoparticles were examined using scanning electron microscopy (SEM) (JSM-IT 100). In order to prepare SEM specimens, gold coatings were applied because gold conducts heat and electricity and is a good infrared refractor [42].

### 4.5. In Vitro Studies

#### Antibacterial and Antifungal Analysis

The agar well diffusion method was used for antibacterial and antifungal analysis that was developed by Vijayan et al. (2018). The antibacterial and antifungal activity of synthesized sulfur nanoparticles was tested against Gram-positive bacteria (*Staph. aureus* and *Bacillus*), Gram-negative bacteria (*E. coli* and *Bordetella*), and fungal strains (*Candida albicans*) [44]. In sterilized Petri dishes, nutrient agar was poured and solidified overnight. Cotton buds were used to spread clinical cultures of *Staph. aureus*, *Bacillus*, *E. coli*, *Bordetella*, and *Candida albicans* all over the solidified agar plates. Both SNPs alone and in combination with antibiotics were evaluated for their effectiveness. The strain of Gram-positive bacteria (*Staph. aureus* and *Bacillus*) was treated with sulfur nanoparticles (SNPs), erythromycin thiocyanate (ET), and a combination of sulfur nanoparticles (SNPs) with erythromycin thiocyanate (SNPsET). The strain of Gram-negative bacteria (*E. coli* and *Bordetella*) were treated with sulfur nanoparticles (SNPs), neomycin sulfate (NS), and a combination of sulfur nanoparticles (SNPs) with neomycin sulfate (SNPsNS). The fungal strain (*Candida albicans*) was treated with sulfur nanoparticles (SNPs), Amphotericin B (AB), and a combination of sulfur nanoparticles with Amphotericin B (SNPsAB). A sterile cork-borer was used to create three wells of 6 mm in each plate. Distilled water was used to disperse nanoparticles. Sulfur nanoparticles were sonicated for twenty minutes at room temperature after being vortexed for five minutes. Sulfur nanoparticles were concentrated in water at 8 mg/mL. Sterile micropipettes were used to administer samples (sulfur nanoparticles, antibiotics, and antibiotics combined with sulfur nanoparticles) into wells. The antibiotics were treated with 20 L of sulfur nanoparticles. The measurement of inhibition zones around each well followed a 24 h incubation period at 37 °C.

### 4.6. Formation of Nanohydrogel

Five different nanohydrogel formulations were prepared. The optimal formulation for creating hydrogels and incorporating sulfur nanoparticles into gel to create nanohydrogels was ultimately chosen as F4 (Table 3). By scattering 8.5 g of carbopol-934 in 85 mL of distilled water and thoroughly mixing it for 15 min at a speed of 10,000 rpm with a magnetic stirrer, nanohydrogel was formed. After that, 1.5 mL of triethanolamine, a cross-linking agent, was added to the gel. Then, hydrogel was subjected to a 5-min sonication process to eliminate the entrapped air bubbles. The hydrogel was then wrapped in aluminum foil and left in the dark overnight to fully cross-link the polymeric gel with TEA and allow it to swell completely. Nanohydrogel was created by adding 5 g of sulfur nanoparticles and homogenizing them for 3 min with a magnetic stirrer (Table 4) [45].

### 4.7. Characterization of Nanohydrogel

#### 4.7.1. pH of Nanohydrogel

The pH values of nanohydrogel samples were determined using a digital pH meter (InoLab^®^, Bremen, Germany) The values were recorded in triplicate; mean ± SD, *n* = 3 [46].

#### 4.7.2. Homogeneity Test

To evaluate the formulations’ compositions, a homogeneity test was performed for optimized nanohydrogel formulation (NHGF4). NHGF4 homogeneity was visually evaluated. Homogeneity was performed to evaluate how the optimized formulation (NHGF4) would feel and seem in usage [47].

#### 4.7.3. Viscosity of Nanohydrogel

An NDV-Series viscometer (Moderner, Shanghai, China) was used to measure the viscosity of optimized nanohydrogel formulation (NHGF4). In the viscometer, spindle number 2 rotated at 6, 12, 30, and 60 revolutions per minute. Samples were measured at a temperature of 30 °C. The data were collected in triplicate with means ± SD, *n* = 3 [48].

### 4.8. Skin Irritation Test

Tests were conducted to determine the effectiveness of an optimized nanohydrogel formulation (NHGF4). A NOC for the project was approved by the Gomal Centre of Pharmaceutical Science, Faculty of Pharmacy, Gomal University, Dera Ismail Khan, KP, Pakistan. In this study, healthy albino male rats weighing 250 g were used. Food was provided as per the standard recommendations [49]. During the experiment, rats were kept at room temperature (25 ± 2 °C), and humidity was maintained at 50 ± 10%. A total of two groups of rats were used (Group I and Group II). As a control, rats in Group I were treated with the standard irritant formalin, whereas in Group II, rats were treated with prepared nanohydrogel formulations (NHGF4). Skin irritation was scored visually based on the appearance of the skin. There were four categories of skin irritation: “0” means there is no skin irritation, “1” means there is a slight irritation, “2” means there is a well-defined irritation, “3” means there is a moderate irritation, and “4” means there is scarring [49].

### 4.9. In Vivo Studies

The in vivo study was approved by the Ethical Review Board, Gomal University, Dera Ismail Khan, KP, Pakistan (protocol code No:332/ERB/GU and 16 November 2022). The in vivo studies were conducted on healthy male rats weighing 250 g. Standard food was given to the rats chosen for the in vivo study. Rats were fed 10% white fish meat, 40% bran, 18% middling, 20% grass meal, and water, as necessary. Rats were kept at room temperature (25 ± 2 °C), with 50 ± 10% relative humidity. A total of two groups of rats (Group I and Group II) were studied. In Group I, rats were left untreated and served as negative controls, while in Group II, rats were treated with nanohydrogel formulation (NHGF4) and served as experimental groups. The anesthesia was administered using a ketamine and xylazine injection solution. An electric trimmer was used to shave and clean the backs of the rats after they had been anesthetized. One cage was kept per rat to prevent rats from injuring one another. Biopsy punches were used to create 8 mm-deep wounds in this area. A nanohydrogel formulation (NHGF4) was administered to Group II rats instead of Group I rats, which were left untreated. On days 0, 7, and 14, rats’ wounds were evaluated and snapped at the same height and standing position. To measure wound closure, a scale was used. A total of three samples were collected, each in triplicate [49].

### 4.10. Stability Test

Phase separation, color change, and odor changes were considered in this test’s execution. The shelf life and color change were assessed through visual examination of the SNPs-embedded nanohydrogel formulation (NHGF4) across various time periods. NHGF4 was diluted with distilled water to check its stability at low and exaggerated temperatures, i.e., 4 ± 2 °C and 40 ± 2 °C, respectively. Phase separation was assessed by allowing the sample to remain in a water bath at 80 °C for 1 h [50].

### 4.11. Statistical Analysis

The data were analyzed using Statistical Package Minitab^®^ version 20 (Minitab, LLC, State College, PA, USA) and reported as a Kolmogorov–Smirnov (K–S) test before applying one-way ANOVA. It was statistically analyzed using ANOVA (one-way analysis of variance), with post hoc analysis by Tukey HSD test (IBM^®^ SPSS^®^ Statistics version 19, Armonk, New York, NY, USA). *p* < 0.05 was considered statistically significant. Data were reported in triplicate (*n* = 3), and mean ± SD.

## Figures and Tables

**Table 1:** Organoleptic analysis of prepared NHGF4.

F. Code	pH	Odor	Color	Phase Separation	Homogeneity	Consistency
NHGF4	5.5 ± 0.63	Very Good	Off−white	Nil	Excellent	Very Good

**Table 2:** Storage stability of NHGF4 at refrigerated and accelerated temperatures for 60 days.

Parameters	Temperature	
	4 ± 2 °C	40 ± 2 °C
Odor	No change	No change
Color	Off white	Off white
Phase separation	Nil	Nil
Homogeneity	Excellent	Excellent
Consistency	Very Good	Very Good

**Table 3:** Composition of hydrogel (*w*/*w*).

S. No	Ingredients	F1 (g)	F2 (g)	F3 (g)	F4 (g)	F5 (g)
1	Carbopol-934	19	15	10	8.5	5
2	Distilled water	80	83	87.5	90	92
3	Triethanolamine (TEA)	1	2	2.5	1.5	3
4	Total	100	100	100	100	100

Note: F1−F5 (Formulation F1−Formulation F5).

**Table 4:** Loading of sulfur nanoparticles (*Citrus limon*) in optimized hydrogel formulation.

S. No	Ingredients	Nanoparticle Loaded Nanohydrogel (NHGF4)
1	Sulfur nanoparticles (*Citrus limon*)	5 g
2	Carbopol-934	8.5 g
3	Distilled water	85 g
4	Triethanolamine (TEA)	1.5 g

Note: NHGF4 (Nanohydrogel Formulation 4).

## Data Availability

Not applicable.
